# Supplementary material for: Assessment of dynamic cerebral autoregulation in humans: Is reproducibility dependent on blood pressure variability?
Source: PLoS One. 2020 Jan 10;15(1):e0227651. doi: 10.1371/journal.pone.0227651 (PMC6954074; doi:10.1371/journal.pone.0227651)
Supplement: S1 Table — Category: 1 = TFA-like methods, 2 = ARI-like methods, 3 = correlation-like methods, Method group: 1 = TFA, 2 = Laguerre expansions, 3 = Wavelets, 4 = IR-filter, 5 = ARX, 6 = ARI, 7 = ARMA-ARI/ARX, 9 = IR-filter, 10 = correlation coefficient; VLF: very low frequency; LF: low frequency; BP: blood pressure; FFT: fast Fourier transform; ARI: autoregulation index; ARX: autoregressive model with exogenous input; Centre names are listed in S2 Table. (DOCX) [file pone.0227651.s001.docx]

**S1 table.** **S1 table: Analysis Methods with references and corresponding output variables per center**

| **Centre**  **number** | **Method** | **Output Variables** | **Category** | **Method group** | **Reference** |
| --- | --- | --- | --- | --- | --- |
| 1 | 1.1 Transfer Function Analysis  1.2 Autoregulation index | Coherence, Gain (cm/s/mmHg) and Phase (rad) in VLF, LF  ARI | 1  2 | 1  6 | ^1^  ^2^ |
| 2 | 2.1 Laguerre expansion of 1^st^-order Volterra kernels, single input (BP)  2.2 Laguerre expansion of 1^st^-order Volterra kernels, dual input (BP, CO_2_) | Gain (cm/s/mmHg) and Phase (rad) in VLF, LF  Gain (cm/s/mmHg) and Phase (rad) in VLF, LF | 1  1 | 2  2 | ^3,4,5,6^ |
| 3 | 3.1 Transfer Function Analysis  3.2 Transfer Function Analysis | Coherence, Gain (cm/s/mmHg), Phase (rad) in VLF, LF  Coherence, Gain (%/%) in VLF, LF | 1  1 | 1  1 | ^1^ |
| 4 | 4.1 Autoregulation index (FFT)  4.2 Autoregulation index (Moving Average 1)  4.3 Autoregulation index (Moving Average 2) | ARI  ARI  ARI | 2  2  2 | 6  7  7 | ^2,7^ |
| 5 | 5.1 Transfer Function Analysis  5.2 Oblique and Orthogonal Subspace Projections | Coherence, Gain (cm/s/mmHg), Phase (rad) in VLF, LF  Subspace Ratio’s | 1  3 | 1  10 | ^1^  ^8^ |
| 6 | 6.1 Transfer Function Analysis | Coherence, Gain (cm/s/mmHg), Phase (rad) in VLF, LF | 1 | 1 | ^9,10^ |
| 7 | 7.2 Transfer Function Analysis | Coherence, Gain (cm/s/mmHg), Phase (rad) in VLF, LF | 1 | 1 | ^11^ |
| 8 | 8.1 ARX  8.2 Wavelet Analysis | ARX Coefficient (3rd)  Synchronisation index, Phase (rad) in VLF,LF | 2  1 | 7  3 | ^7,12,13^  ^14^ |
| 9 | 9.1 Transfer Function Analysis  9.2 Convergent cross mapping | Coherence, Gain (cm/s/mmHg), Phase (rad) in VLF, LF  CCM correlation coefficient | 1  3 | 1  10 | ^15,16^  ^17^ |
| 11 | 11.1 Transfer Function Analysis,  11.2 Transfer Function Analysis  11.3 Transfer Function Analysis  11.4 Univariate Transfer Function Analysis (parametric method)  11.5 Univariate Impulse Response  (parametric method)  11.6 Multivariate Transfer Function Analysis (parametric method) | Coherence, Gain (cm/s/mmHg), Phase (rad) in VLF, LF  Coherence, Gain (%/mmHg), Phase (rad) in VLF, LF  Coherence, Gain (%/%) in VLF, LF  Coherence, Gain (%/%), Phase (rad) in LF  The second filter coefficient (h_1_) of the estimated FIR  Gain (%/%) and Phase (rad) for LF band | 1  1  1  1  2  1 | 1  1  1  4  9  4 | ^18,19^ |
| 12 | 12.1 Transfer Function Analysis  12.2 Autoregulation index  12.3 Wavelet Coherence Analysis | Coherence, Gain (cm/s/mmHg), Phase (rad) in VLF, LF  ARI  Gain (cm/s/mmHg) and Phase (rad) in VLF, LF | 1  2  1 | 1  6  3 | ^1^  ^2^  ^20,21^ |
| 13 | 13.1 Transfer Function Analysis | Coherence, Gain (cm/s/mmHg), Phase (rad) in VLF, LF | 1 | 1 | ^22^ |
| 14 | 14.1 ARX models: 1 input  14.2 ARX models: 2 inputs  14.3 Laguerre expansion FIR models, single input (BP)  14.4 Laguerre expansion FIR models, dual input (BP, CO_2_)  14.5 Transfer function analysis | Gain (cm/s/mmHg), Phase (rad) in VLF, LF  Gain (cm/s/mmHg), Phase (rad) in VLF, LF  Gain (cm/s/mmHg), Phase (rad) in VLF, LF  Gain (cm/s/mmHg), Phase (rad) in VLF, LF  Coherence, Gain (cm/s/mmHg), Phase (rad) in VLF, LF | 1  1  1  1  1 | 5  5  2  2  1 | ^23,24^  ^25,26^  ^27^ |

Category: 1= TFA-like methods, 2= ARI-like methods, 3= correlation-like methods, Method group: 1=TFA, 2=Laguerre expansions, 3=Wavelets, 4=IR-filter, 5=ARX, 6=ARI, 7=ARMA-ARI/ARX, 9=IR-filter, 10=correlation coefficient; VLF: very low frequency; LF: low frequency; BP: blood pressure; FFT: fast Fourier transform; ARI: autoregulation index; ARX: autoregressive model with exogenous input; Centre names are listed in Table S2.

**References for Analysis Methods**

1 Zhang R, Zuckerman JH, Giller CA, Levine BD. Transfer function analysis of dynamic cerebral autoregulation in humans. *Am J Physiol* 1998; **274**: H233-41.

2 Panerai RB, White RP, Markus HS, Evans DH. Grading of Cerebral Dynamic Autoregulation From Spontaneous Fluctuations in Arterial Blood Pressure. *Stroke* 1998; **29**: 2341–2346.

3 Marmarelis VZ. *Nonlinear dynamic modeling of physiological systems*. 2004 doi:10.1002/9780471679370.

4 Marmarelis VZ, Shin DC, Orme ME, Zhang R. Model-based physiomarkers of cerebral hemodynamics in patients with mild cognitive impairment. *Med Eng Phys* 2014; **36**: 628–637.

5 Marmarelis VZ, Shin DC, Orme ME, Zhang R. Model-based quantification of cerebral hemodynamics as a physiomarker for Alzheimer’s disease? *Ann Biomed Eng* 2013; **41**: 2296–2317.

6 Marmarelis VZ, Shin DC, Orme M, Zhang R. Time-varying modeling of cerebral hemodynamics. *IEEE Trans Biomed Eng* 2014; **61**: 694–704.

7 Panerai RB, Eames PJ, Potter JF. Variability of time-domain indices of dynamic cerebral autoregulation. In: *Physiological Measurement*. 2003, pp 367–381.

8 Caicedo A, Varon C, Hunyadi B, Papademetriou M, Tachtsidis I, Van Huffel S. Decomposition of near-infrared spectroscopy signals using oblique subspace projections: Applications in brain hemodynamic monitoring. *Front Physiol* 2016; **7**. doi:10.3389/fphys.2016.00515.

9 Müller M, Österreich M. A comparison of dynamic cerebral autoregulation across changes in cerebral blood flow velocity for 200 seconds. *Front Physiol* 2014; **5 AUG**. doi:10.3389/fphys.2014.00327.

10 Müller M, Bianchi O, Erülkü S, Stock C, Schwerdtfeger K. Changes in linear dynamics of cerebrovascular system after severe traumatic brain injury. *Stroke* 2003; **34**: 1197–1202.

11 Gommer ED, Shijaku E, Mess WH, Reulen JPH. Dynamic cerebral autoregulation: Different signal processing methods without influence on results and reproducibility. *Med Biol Eng Comput* 2010; **48**: 1243–1250.

12 Liu Y, Allen R. Analysis of dynamic cerebral autoregulation using an ARX model based on arterial blood pressure and middle cerebral artery velocity simulation. *Med Biol Eng Comput* 2002; **40**: 600–605.

13 Liu Y, Birch AA, Allen R. Dynamic cerebral autoregulation assessment using an ARX model: Comparative study using step response and phase shift analysis. *Med Eng Phys* 2003; **25**: 647–653.

14 Peng T, Rowley AB, Ainslie PN, Poulin MJ, Payne SJ. Wavelet phase synchronization analysis of cerebral blood flow autoregulation. *IEEE Trans Biomed Eng* 2010; **57**: 960–968.

15 van Beek AHEA, Lagro J, Olde-Rikkert MGM, Zhang R, Claassen JAHR. Oscillations in cerebral blood flow and cortical oxygenation in Alzheimer’s disease. *Neurobiol Aging* 2012; **33**: 428.e21-428.e31.

16 van Beek AHEA, Olde Rikkert MGM, Pasman JW, Hopman MTE, Claassen JAHR. Dynamic Cerebral Autoregulation in the Old Using a Repeated Sit-Stand Maneuver. *Ultrasound Med Biol* 2010; **36**: 192–201.

17 Heskamp L, Abeelen ASSM den, Lagro J, Claassen JAHR. Convergent cross mapping: a promising technique for cerebral autoregulation estimation. *Int J Clin Neurosci Ment Heal* 2016; : S20.

18 Simpson DM, Panerai RB, Evans DH, Naylor AR. Parametric approach to measuring cerebral blood flow autoregulation from spontaneous variations in blood pressure. *Ann Biomed Eng* 2001; **29**: 18–25.

19 Panerai RB, Simpson DM, Deverson ST, Mahony P, Hayes P, Evans DH. Multivariate dynamic analysis of cerebral blood flow regulation in humans. *IEEE Trans Biomed Eng* 2000; **47**: 419–423.

20 Program AS, Sciences O. Interdecadal changes in the ENSO-monsoon system. *J Clim* 1999; : 2679–2690.

21 Grinsted A, Moore JC, Jevrejeva S. Application of the cross wavelet transform and wavelet coherence to geophysical time series. *Nonlinear Process Geophys* 2004; **11**: 561–566.

22 Panerai RB, Rennie JM, Kelsall AWR, Evans DH. Frequency-domain analysis of cerebral autoregulation from spontaneous fluctuations in arterial blood pressure. *Med Biol Eng Comput* 1998; **36**: 315–322.

23 Mitsis GD, Zhang R, Levine BD, Marmarelis VZ. Modeling of Nonlinear Physiological Systems with Fast and Slow Dynamics. II. Application to Cerebral Autoregulation. *Ann Biomed Eng* 2002; **30**: 555–565.

24 Mitsis GD, Zhang R, Levine BD, Tzanalaridou E, Katritsis DG, Marmarelis VZ. Autonomic neural control of cerebral hemodynamics. *IEEE Eng Med Biol Mag* 2009; **28**: 54–62.

25 Mitsis GD, Poulin MJ, Robbins PA, Marmarelis VZ. Nonlinear modeling of the dynamic effects of arterial pressure and CO 2 variations on cerebral blood flow in healthy humans. *IEEE Trans Biomed Eng* 2004; **51**: 1932–1943.

26 Kostoglou K, Debert CT, Poulin MJ, Mitsis GD. Nonstationary multivariate modeling of cerebral autoregulation during hypercapnia. *Med Eng Phys* 2014; **36**: 592–600.

27 Meel-van den Abeelen ASS, Simpson DM, Wang LJY, Slump CH, Zhang R, Tarumi T *et al.* Between-centre variability in transfer function analysis, a widely used method for linear quantification of the dynamic pressure-flow relation: The CARNet study. *Med Eng Phys* 2014; **36**: 620–627.
