# Supplementary material for: Assessment of dynamic cerebral autoregulation in humans: Is reproducibility dependent on blood pressure variability?
Source: PLoS One. 2020 Jan 10;15(1):e0227651. doi: 10.1371/journal.pone.0227651 (PMC6954074; doi:10.1371/journal.pone.0227651)
Supplement: S2 Table — (DOCX) [file pone.0227651.s002.docx]

| **Name** | **Institution** | **Country** | **Role** | **Centre**  **Number** |
| --- | --- | --- | --- | --- |
| E Borg-Seng-Shu  RC Nogueira | Department of Neurology, Hospital das Clinicas  University of Sao Paulo | Brazil | Analysis | 1 |
| VZ Marmarelis  DC Shin | Department of Biomedical Engineering  University of Southern California, Los Angeles | USA | Analysis | 2 |
| R Zhang  T Tarumi | IEEM, Presbyterian Hospital Dallas  University of Texas Southwestern Medical Center | USA | Analysis  Data Provider | 3 |
| RB Panerai | Department of Cardiovascular Sciences  University of Leicester | UK | Analysis  Data Provider  Trial Coordination | 4 |
| S van Huffel  A Caicedo | Department of Electronic Engineering (ESAT), STADIUS Center for Dynamical Systems, Signal Processing and Data Analytics, KU Leuven, Belgium; imec | Belgium | Analysis | 5 |
| M Müller | Department of Neurology  Luzerner Kantonsspital | Switzerland | Analysis | 6 |
| ED Gommer | Department of Clinical Neurophysiology  University Hospital Maastricht | Netherlands | Analysis  Data Provider | 7 |
| SJ Payne  A Mahdi | Department of Engineering Science  University of Oxford | UK | Analysis | 8 |
| JAHR Claassen  ML Sanders | Department of Geriatric Medicine  Radboud University Nijmegen | Netherlands | Analysis  Data Provider  Trial Coordination | 9 |
| DM Simpson  D Nikolic | Institute of Sound and Vibration Research  University of Southampton | UK | Analysis  Data Provider | 11 |
| JWJ Elting  M Aries | Department of Neurology  University Medical Center Groningen | Netherlands | Analysis  Data Provider  Trial Coordination | 12 |
| C Puppo  B Yelicich | Departamento de Emergencia, Hospital de ClínicasUniversidad de la República, Montevideo | Uruguay | Analysis | 13 |
| GD Mitsis  K Kostoglou | Department of Bioengineering  Department of Electrical, Computer and Software Engineering  McGill University, Montreal | Canada | Analysis | 14 |

**S2 Table.** Pa**rticipating centres and their roles**

Centre 10 withdrew their results from analysis because their method was superseded by recent developments that would disadvantage their original approach.
