# Supplementary material for: Assessment of dynamic cerebral autoregulation in humans: Is reproducibility dependent on blood pressure variability?
Source: PLoS One. 2020 Jan 10;15(1):e0227651. doi: 10.1371/journal.pone.0227651 (PMC6954074; doi:10.1371/journal.pone.0227651)
Supplement: S1 File — (DOCX) [file pone.0227651.s003.docx]

**Monte Carlo Simulation details:**

DCA analysis methods results were simulated by creating correlated random data arrays, with one random data array representing the results of one DCA analysis method. Seventy-five data points were generated per data array. Between DCA analysis method correlation coefficients were obtained from the artificial data, and were used to calculate correlation and variance-covariance matrices. Two instances of the TSA Random Sequence Generation VI application in labview 2014 were used to generate the correlated random data arrays twice. ICC values were calculated between the first and second data arrays for each simulated DCA method and the mean ICC, the variance of the ICC values and all between method ICC differences were calculated. This process was repeated 10.000 times to obtain a distribution of variance ICC values, from which the 95th percentile was used as a cut-off value to define a statistically significant overall difference in ICC between analysis methods. For Post Hoc tests, the mean and standard deviation of the simulated between method ICC differences was used in a cumulative distribution function, to define 2-sided probability values for the observed between method ICC differences. Due to the high number of Post Hoc comparisons, the probabilities were corrected for multiple comparisons by implementing the Benjamini-Hochberg correction procedure, with a false positive discovery rate of 10%. Finally, a Post Hoc sum-score was constructed: for any analysis method, a positive significant difference with another analysis method was scored as +1, a negative significant difference as -1 and no significant difference as 0. The sum-score is calculated as the sum of all difference scores with all other analysis methods present in the analysis. Differences in ICC values between individual analysis methods were investigated within broad categories of analysis methods only, and not between different categories.
