# Supplementary material for: Assessment of dynamic cerebral autoregulation in humans: Is reproducibility dependent on blood pressure variability?
Source: PLoS One. 2020 Jan 10;15(1):e0227651. doi: 10.1371/journal.pone.0227651 (PMC6954074; doi:10.1371/journal.pone.0227651)
Supplement: S1 Fig — Median lowest PSD-MABP levels for subjects with and without vasoactive medication, in all frequency bands (VLF, LF and HF). No significant differences were found between the two groups. (DOCX) [file pone.0227651.s004.docx]

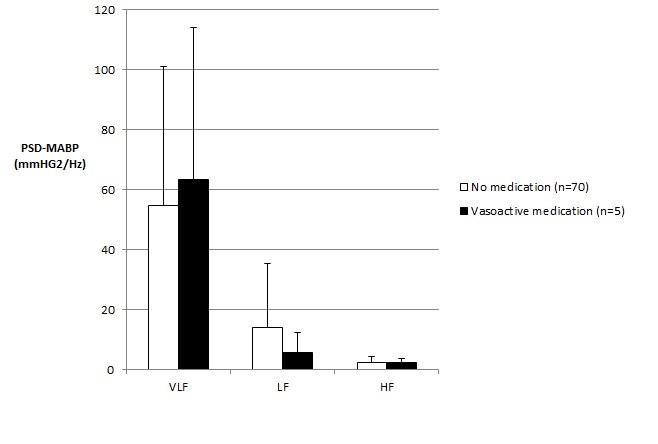


**Figure S4.**. PSD-MABP comparison between subjects with and without vasoactive medication.

Median lowest PSD-MABP levels for subjects with and without vasoactive medication, in all frequency bands (VLF, LF and HF). No significant differences were found between the two groups.
