# Supplementary material for: Assessment of dynamic cerebral autoregulation in humans: Is reproducibility dependent on blood pressure variability?
Source: PLoS One. 2020 Jan 10;15(1):e0227651. doi: 10.1371/journal.pone.0227651 (PMC6954074; doi:10.1371/journal.pone.0227651)
Supplement: S2 Fig — Beeswarm letter-boxplot with ICC values for Gain LF (upper figure) and Gain VLF (lower figure) for different cut-off levels of PSD-MABP. Each analysis method is represented by a letter. No significant differences between methods were found. (DOCX) [file pone.0227651.s005.docx]

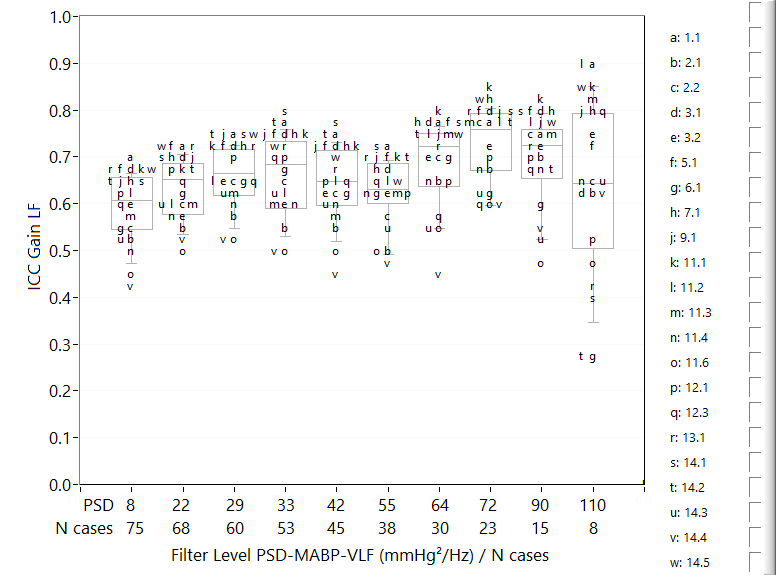


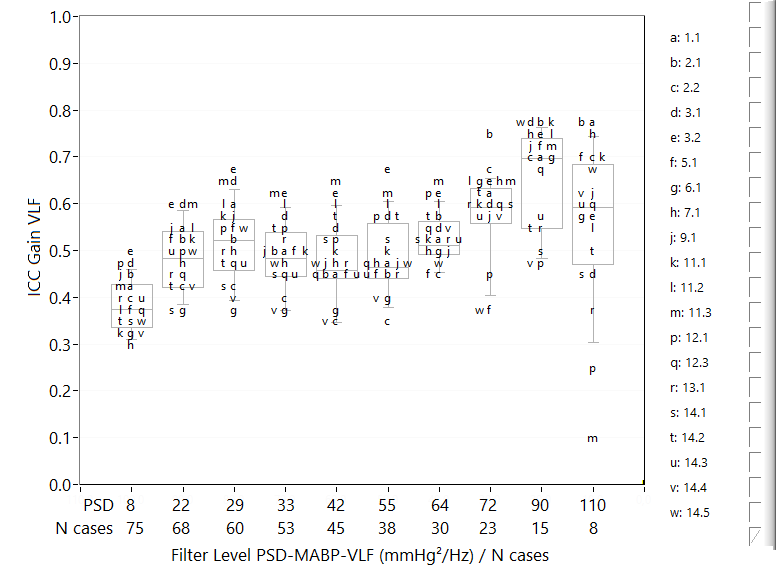


**Figure S1.** Beeswarm letter-boxplot with ICC values for Gain LF (upper figure) and Gain VLF (lower figure) for different cut-off levels of PSD-MABP. Each analysis method is represented by a letter. No significant differences between methods were found.
