# Supplementary material for: Assessment of dynamic cerebral autoregulation in humans: Is reproducibility dependent on blood pressure variability?
Source: PLoS One. 2020 Jan 10;15(1):e0227651. doi: 10.1371/journal.pone.0227651 (PMC6954074; doi:10.1371/journal.pone.0227651)
Supplement: S4 Fig — Beeswarm letter-boxplot with ICC values for ARI and correlation like indices for different cut-off levels of PSD-MABP. Each analysis method is represented by a letter. ! :indicates cut-off level at which significant differences between the methods were found. Post hoc sum-scores for each cut-off level with significant differences between the methods are indicated in the legend, from left to right. For each method, a significant positive ICC difference with another method is scored as +1, no difference as 0, and a negative difference as -1. The sum of all the scores in the post-hoc sum-score. Negative ICC values do not appear in this plot. H and I indicate correlation like indices, which were not included in calculation of the boxplot or in the statistical analysis. (DOCX) [file pone.0227651.s007.docx]

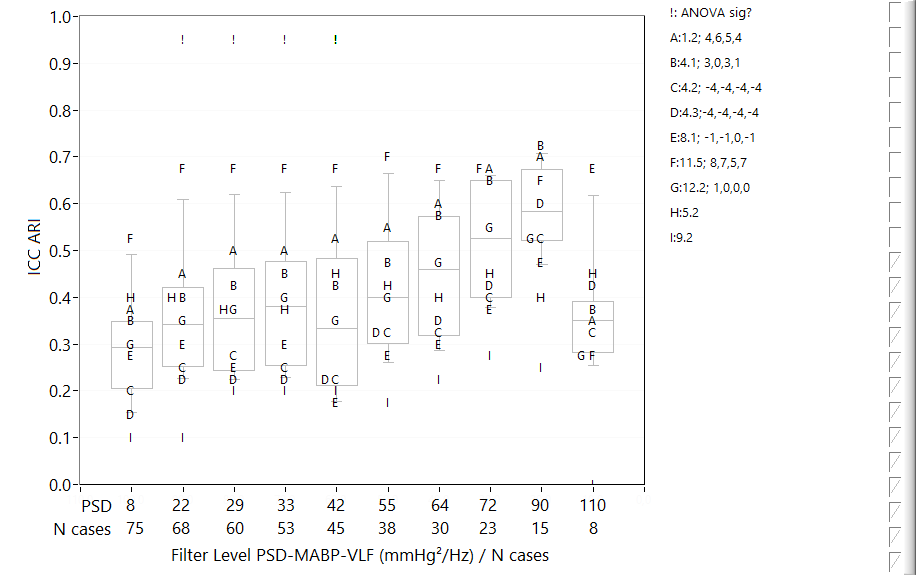


**Figure S3.** Beeswarm letter-boxplot with ICC values for ARI and correlation like indices for different cut-off levels of PSD-MABP. Each analysis method is represented by a letter. ! :indicates cut-off level at which significant differences between the methods were found. Post hoc sum-scores for each cut-off level with significant differences between the methods are indicated in the legend, from left to right. For each method, a significant positive ICC difference with another method is scored as +1, no difference as 0, and a negative difference as -1. The sum of all the scores in the post-hoc sum-score. Negative ICC values do not appear in this plot. H and I indicate correlation like indices, which were not included in calculation of the boxplot or in the statistical analysis.
