# Supplementary material for: Assessment of dynamic cerebral autoregulation in humans: Is reproducibility dependent on blood pressure variability?
Source: PLoS One. 2020 Jan 10;15(1):e0227651. doi: 10.1371/journal.pone.0227651 (PMC6954074; doi:10.1371/journal.pone.0227651)
Supplement: Attachment — Submitted filename: Reply_reviewers_Elting_et_al.docx [file pone.0227651.s009.docx]

*We want to thank the reviewer for his/her useful comments*

Reviewer #1: This manuscript provides us the novel clinical implications in the assessment of cerebral circulation. The methods were reasonable, and discussion is quite scientific.

To strength the manuscript I would like to ask several minor questions to the authors.

1) How did you calculate the sample size (75 healthy subjects)?

*This was determined arbitrarily, i.e. no formal power calculation for the ICC statistic was performed. Although methods do exist to perform a power calculation for ICC analysis in general (ref: power calculation for the likelihood ratio-test when comparing two dependent intraclass correlation coefficients. Giraudeau et. Al. Computer methods and Programs in Biomedicine 2005. 77. 165-173), they cannot be easily generalized to more complex ICC comparisons.*

*Another option would have been to use a Monte Carlo simulation to perform a power analysis. However, for that analysis to be valid, the between method correlation coefficients have to be known. These were not known before the trial started, which prevented us from conducting a valid power analysis.*

*In a previous study, Brodie et al (Clin Sci 2009) provided estimates for the sample size in studies using the ARI index, but similar information was not available for the other indices in our study. From the information provided, with n=75 subjects in our case, we can estimate that a difference of ARI=0.77 could be detected with 80% power at α =0.05, which should be very satisfactory to detect intra-subject differences (Brodie et al Clin Sci 2009).*

*This is why we were unable to do a formal power analysis for all indices before the trial started.*

*A sentence was added to the methods section:*

A formal sample size calculation was not possible before the study was performed, due to the lack of information about inter-subject variability for the majority of autoregulation indices. In a previous study, Brodie et al (Clin Sci 2009) provided estimates for the sample size in studies using the ARI index, but similar information was not available for the other indices in our study. From the information provided, with n=75 subjects in our case, we can estimate that a difference of ARI=0.77 could be detected with 80% power at α =0.05, which should be very satisfactory to detect intra-subject differences (Brodie et al Clin Sci 2009).

2) As described in the limitation, I am also afraid the effects of hypertensive agents to your results. Your discussion about this issue in the limitation paragraph was quite reasonable. If possible, it will be helpful to us that the subjects with hypertensive agents would be excluded or showed in separate table.

*A separate figure with the data of subjects who were on hypertensive drugs was constructed. There were no significant differences in lowest PSD-MABP between the vasoactive medication group and the rest of the subjects in all frequency bands. A sentence and reference to the figure was added to the Results and Discussion section.*

*Results:*

Median lowest PSD-MABP was comparable between those that used vasoactive medication and those without any medication (S4 fig.)

*Discussion:*

We cannot exclude the possibility that low PSD-MABP power was in part due to the use of medication, but suspect that it is likely that this is not a major cause, since median lowest PSD-MABP was comparable between those that used vasoactive medication and those without any medication (S4 fig.)

3) I would like you to show more concrete clinical perspectives.

We have added a section: clinical perspective and recommendations in the discussion section. We added the following sentences:

Based on the results in this study, it seems advisable to calculate PSD-MABP in the VLF and LF bands during data acquisition, so that one can evaluate if sufficient power is present in the MABP signal.

And:

If not sufficient, recordings may be extended while selectively leaving out data segments with low PSD-MABP. Alternatively, oscillation inducing maneuvers can be attempted. In clinical situations, longer measurement duration without loss of data quality may sometimes be difficult, for example in acute stroke patients who may suffer from delirium and restlessness. On the other hand, in the intensive care setting when patients are sedated and intubated, a longer measurement duration may be more easily obtained.

*We want to thank the reviewer for his/her useful comments*

Reviewer #2: The reproducibility of dynamic cerebral autoregulation assessment is an important step for its clinical use. The authors found that the assessment was not reproducible unless MAP variations during the measurement window are significant. I found the analysis in the paper is sound. I have the following suggested revisions:

1. The authors mention that reproducibility will probably improve if measurement duration is increased. This is based on their use of the Spearman-Brown prophecy formula. The authors cite a reference for this, but they should also write what the formula is in the manuscript to make it easier to understand Fig. 5. Are longer durations more likely to be reproducible because there are more likely to be significant spontaneous MAP variations?

*The formula was added in the text, methods section. We added:*

The Spearman-Brown calculated ICC (ICC^SB^) is calculated as:

ICC^SB^ $= \frac{nICC}{1+\left( n-1 \right)ICC}$ where ICC is the ICC value based on 2 measurements of 5 minutes and *n* is the factor by which the measurement duration will be extended.

*The confidence limits of TFA directly depend on the number of averages used in the calculations, resulting in smaller confidence limits with longer duration. This will lead to better reproducibility by itself, even without an increase in spontaneous MAP variations. A sentence is already present in the discussion to explain this.*

For example, with TFA, it is known that the confidence limits of the estimates depend on the level of coherence, and on the number of degrees of freedom used in the estimate which is determined by measurement duration and spectral smoothing[34]^,^[35].

We added:

With increasing measurement duration, confidence limits of DCA estimates will be reduced, leading to less variability and higher reproducibility.

2. I would appreciate practical recommendations in the manuscript about what the power spectral density MABP levels need to be for reproducible results.

*We agree with this suggestion, which was also suggested by reviewer 1. We have added a section: clinical perspective and recommendations in the discussion section. There were already a few sentences on recommended levels for PSD-MABP present in the discussion:*

It is advisable to have at least 29 mmHg^2^/Hz PSD in the VLF when analyzing gain and phase and also ARI, so that at least moderate reproducibility can be achieved for some methods, with good reproducibility for the best performing methods. This will be accompanied by a 20% loss of analyzable cases, if one extrapolates from the relatively large sample adopted in this study. When phase LF is of interest, at least 10 mmHg^2^/Hz PSD is advisable, which will lead to 30% loss of cases, but these estimates need further confirmation in different measurement conditions and patient sub-groups.

*We added the following sentences:*

Based on the results in this study, it seems advisable to calculate PSD-MABP in the VLF and LF bands during data acquisition, so that one can evaluate if sufficient power is present in the MABP signal.

And:

If not sufficient, the recordings may be extended while selectively leaving out data segments with low PSD-MABP. Alternatively, oscillation inducing maneuvers can be attempted. In clinical situations, longer measurement duration without loss of data quality may sometimes be difficult, for example in acute stroke patients who may suffer from delirium and restlessness. On the other hand, in the intensive care setting when patients are sedated and intubated, a longer measurement duration may be more easily obtained.

*We want to thank the reviewer for his/her useful comments*

Reviewer #3: The article "Assessment of dynamic cerebral autoregulation in humans: is reproducibility dependent on blood pressure variability?" by Elting et. al. is an interesting and potentially significant article on the intra-subject variability of cerebral autoregulation measurements. The authors have performed a retrospective analysis of data from several sites, comprising of recordings of mean arterial blood pressure and cerebral blood flow velocity (derived from TCD). The goal of the analysis is to quantify the variability in autoregulation indices specifically with respect to blood pressure variability. The analysis methods, statistical techniques used are rigorous, with instructive and explanative figures. The conclusions drawn by the authors are appropriate. However, a few minor details reduce the clarity and readability of the article. Those and other comments are listed below.

1. The organization of the data sources is somewhat difficult to understand. The authors provide a list of data sources and analysis performed therein in supplementary tables S1 and S2. While the text states that data was derived form 5 participating centers, the tables seem to suggest 6 data sources.

*The reviewer is correct in noting that there are actually 6 data providing centers. This is an error on our part. We changed this in the methods section.*

Furthermore, it is evident that the tables that each data source is used more than once. However, it is difficult for the reader to ascertain if the same class of analysis (e.g., transfer function) is performed more than once on the same data set. Furthermore, are all three categories of analysis available for all data sources? Some clarity on this, perhaps in the form of a supplementary table that lists the data source and associated analysis, will help.

*A separate study flowchart figure was constructed that states the data sources and analysis methods in a more visual way.*

*We added in the methods section:*

A study flowchart is presented in Fig 1.

2. One of the goals of the analysis is to look at the variabilities of autoregulatory responses at different time points. However, the authors do not specify the range of time points from which dual ratings were derived for analysis (besides from an upper bound of 3 months). The time between measurements may be a significant factor in the variability - this is not considered here.

*The reviewer is right, we did not consider this. Since we only required that the interval between the measurements was less than 3 months, we do not have the data on the exact time difference between the measurements for every data contributing center. Therefore, formal analysis of the influence of time was not possible.*

*we added a remark in the limitation section in the Dicussion to address this point:*

Lastly, we did not consider the influence of time between the two measurements, which could be up to 3 months in this study. We cannot exclude the possibility that a longer interval between the two measurements will introduce extra variability in the DCA estimates.

3. For the surrogate data used to replace data of poor quality, the authors performed simulations based on the Tiecks model. How did the authors know which ARI to use, if the data was of poor quality to perform analysis in the first place? Also, please include a citation for the model.

*We want to apologize for the confusion. We had included a short sentence to the reviewer, which can be found in the same section as the remark on the Tiecks model:*

The results for the artificial and physiological data have been presented elsewhere, and will not be reported here[21]

*The artificial data were not used in the present paper, since they have already been published. Therefore, it is indeed confusing to elaborate further on the details of the data generation. To make this clearer, we chose to delete any references to the Tiecks model, and the way in which the data was generated, but maintained the reference to the study publication. Now, we simply use these remarks to explain why there are a different number of cases with left and right sided measurements.*

4. It might help to describe the outcome variables from the three autoregulation measurement classes.

*This is provided by S1 table: Analysis Methods with references and corresponding output variables per center, with method category, method group and references. If the reviewer means a short explanation of each variable: this will probably result in quite a bit of extra text, because the parameters are sometimes the result of complex mathematical procedures. This will not fit nicely in a table. We feel it is better to provide references for the interested reader.*

5. The authors describe the PSD based case removal approach, but do not provide sufficient justification for why the authors considered PSD as a measure of variability. Similarly, what is the rationale for using the 'lowest' PSD-MABP as the independent variable for correlation analyses?

*We write in the introduction:*

Some studies have shown that DCA parameters are more stable if PSD-MABP is high, and can become variable if PSD-MABP is low[23]^,^[24]. A number of techniques to increase PSD-MABP variability have been described, and in some of these studies reproducibility of the DCA estimates have been shown to improve[23]^,^[25]^,^[26]^,^[27].

*We feel that this justifies the main focus of this larger study, which is to evaluate if DCA reproducibility will increase if subjects with low PSD-MABP are left out of the analysis.*

*The rationale for using the lowest PSD-MABP of the 2 measurements as a selection criterion is as follows:*

*If PSD-MABP is related to reproducibility, i.e. ICC of two measurements, a low ICC can be already be the result of one of the measurements having low PSD-MABP. If one measurement is variable, and the other is not, the result will still be a low reproducibility and a low ICC. Therefore, in order to have a good reproducibility, both measurements should have a high PSD-MABP. This requirement is not necessarily met by mean or median statistics. This can be achieved by demanding a PSD-MABP value that is above a threshold value for the lowest PSD-MABP, then the highest PSD-MABP will automatically also be above the threshold, and both measurements will have high PSD-MABP, resulting in a better reproducibility.*

*We added in the methods section:*

The lowest PSD value was used, since this will assure that when using PSD-MABP as a filter variable based on threshold levels, both PSD-MABP values will be above the threshold.
